# Supplementary material for: Inhibition of Fusarium oxysporum growth in banana by silver nanoparticles: In vitro and in vivo assays
Source: PLoS One. 2025 Feb 24;20(2):e0308200. doi: 10.1371/journal.pone.0308200 (PMC11849816; doi:10.1371/journal.pone.0308200)
Supplement: S1 Table — Inhibition percentages of nine different concentrations of AgNPs against four FOC race 1 strains. The table presents the inhibitory effects observed for each FOC strain at AgNP concentrations ranging from 0 mg L−1 to 100 mg L−1. Values represent the mean percentage inhibition ± standard deviation from three independent experiments with three technical replicates. (PDF) [file pone.0308200.s001.pdf]

S1 Table. Fungal growth inhibition percentages of *Fusarium oxysporum* strains by different concentrations of AgNPs.

AgNPs concentrations [mg L^{-1}]	**Foc Strains**	**AgNPs**		
		**Argovit-1220 Inhibition %**	**Argovit-1221 Inhibition %**	**Argovit-C Inhibition %**
**100**	Ec15	100.00 ±0.00	96.24 ±4.69	99.87 ±0.00
	Ec19	100.00 ±0.00	99.18 ±1.10	99.65 ±0.00
	Ec40	100.00 ±0.00	98.14 ±1.63	99.52 ±0.00
	Ec35	100.00±0.14	84.64 ±8.42	84.26 ±5.89
**50**	Ec15	99.46 ±0.00	95.38 ±4.58	99.31 ±0.00
	Ec19	99.98 ±0.00	98.48 ±1.85	99.80 ±0.00
	Ec40	99.75 ±0.00	96.81 ±2.22	98.84 ±1.15
	Ec35	88.30 ±5.36	74.30 ±16.17	77.06 ±1.70
**25**	Ec15	98.43 ±0.58	86.64 ±3.25	95.56 ±2.31
	Ec19	99.12 ±0.58	95.26 ±4.50	98.03 ±2.31
	Ec40	98.15 ±1.15	87.10 ±9.64	93.75 ±2.08
	Ec35	80.21 ±12.14	60.31 ±14.99	66.62 ±3.43
**12.5**	Ec15	92.77 ±0.00	82.75 ±4.13	89.56 ±3.00
	Ec19	94.90 ±1.00	75.20 ±4.44	93.98 ±3.46
	Ec40	93.06 ±2.08	72.57 ±0.62	89.33 ±5.20
	Ec35	66.28 ±10.92	44.95 ±9.35	55.36 ±5.18
**6.3**	Ec15	82.12 ±3.06	59.66 ±11.59	73.56 ±4.04
	Ec19	82.07 ±4.16	51.24 ±4.29	85.79 ±3.00
	Ec40	78.91 ±8.14	57.28 ±10.08	78.24 ±5.51
	Ec35	48.91 ±8.10	36.84 ±11.88	48.27 ±8.32
**3.1**	Ec15	50.85 ±4.16	43.02 ±16.82	58.64 ±2.00
	Ec19	55.29 ±7.81	34.68 ±3.79	64.43 ±6.08
	Ec40	48.52 ±2.89	39.99 ±11.57	56.17 ±4.93
	Ec35	29.86 ±2.59	28.03 ±9.30	34.41 ±9.27
**1.6**	Ec15	32.68 ±1.73	30.11 ±19.78	40.60 ±1.15
	Ec19	32.02 ±3.06	17.42 ±1.88	48.45 ±6.08
	Ec40	29.04 ±2.52	25.46 ±9.90	38.02 ±1.73
	Ec35	19.87 ±5.65	22.79 ±9.08	25.37 ±7.89
**0.8**	Ec15	15.34 ±1.53	19.11 ±14.23	17.51 ±2.08
	Ec19	14.41 ±3.00	10.08 ±1.73	25.72 ±4.58
	Ec40	19.85 ±5.20	17.83 ±11.99	18.41 ±1.73
	Ec35	11.00 ±7.47	12.01 ±4.42	10.01 ±4.89
**0**	Ec15	0.00 ±0.01	0.00 ±0.01	0.00 ±0.02
	Ec19	0.00 ±0.01	0.00 ±0.01	0.00 ±0.01
	Ec40	0.00 ±0.01	0.00 ±0.01	0.00 ±0.01
	Ec35	0.00 ±0.01	0.00 ±0.00	0.00 ±0.01
