# Supplementary material for: Inhibition of Fusarium oxysporum growth in banana by silver nanoparticles: In vitro and in vivo assays
Source: PLoS One. 2025 Feb 24;20(2):e0308200. doi: 10.1371/journal.pone.0308200 (PMC11849816; doi:10.1371/journal.pone.0308200)
Supplement: Attachment — Submitted filename: Response to Reviewers.pdf [file pone.0308200.s006.pdf]

Guayaquil, 10 – 21 -2024

Dear Adalberto Benavides-Mendoza, Ph.D.,
Academic Editor,
PLOS ONE

We would like to express our gratitude to you and the reviewers for the constructive and insightful comments on our manuscript, titled "Inhibition of *Fusarium oxysporum* growth in banana by silver nanoparticles: in vitro and in vivo assays" (Manuscript ID: PONE-D-24-29951R1). We have carefully considered all the feedback and revised the manuscript accordingly.

Below, we provide detailed responses to each comment from the manuscript and the reviewer's comments to the authors and outline the changes made in the revised manuscript with track changes.

**Reviewer's comments from the manuscript**
**Reviewer 1:**
**Comment 1:**
"comercial?" (line 32)
**Response:**
Thank you for this comment. In response, it has been clarified in the methodology that the AgNPs formulations are commercial, so the following sentence has been added on line 121: "commercial formulations".

**Comment 2:**
"Indicate which strain Race 1, Race 2 or other to avoid confusion." (line 33)
**Response:**
We appreciate this suggestion. In response, it was specified that Fusarium strains are race 1 in the manuscript to avoid confusion. Thus, "race 1" was added in the abstract on lines 33 and 40.

**Comment 3:**
"the study also included an in vitro phase.... reformulate the sentence. Avoid the superlative "the best"" (line 39)
**Response:**
We agree with this suggestion. The revised text on line 39 now reads as: "To our knowledge, this is the first study to test the potential of AgNPs against *Fusarium oxysporum* race 1 in vitro and in vivo under greenhouse conditions."

**Comment 4:**
"Foc Race 4 is not exactly a new race... it is other race nowadys" (line 57)
**Response:**
We agree with this suggestion. In the manuscript, we changed the word "new" to "another" in line 58.

**Comment 5:**
"Not sure the relevance of this information to the paper objectives" (line 115)
**Response:**
We agree with this suggestion. A more summarized table was left with the characteristics of the AgNPs, such as the type of stabilized used, the content in percentage of Ag and of the stabilizer, and characterizations such as the average diameter (nm) and the morphology of the nano. Table 1 now appears on line 128.

**Comment 6:**
"Was it performed three times?.. and each study included three replication per treatment? please clarify." (line 132)
**Response:**
Thank you for the insightful suggestion. To improve clarity, we have restructured the sentence to "The study was performed three times, and each treatment included three replications." on lines 146, and 147.

**Comment 7:**
"one or various?" (line 159)
**Response:**
We agree with this comment and have revised the methodology and clarified that there are two experiments separated by AgNP application methods (foliar and drench), and each of them has two inoculation methods (root dipping and drenching). In the manuscript, we have restructured the methodology on line 174-182 and provided more detailed explanations

**Comment 8:**
"Why? two type of application. Foc is a root fungus." (line 170)
**Response:**
We understand the reviewer's concern. To clarify, the decision to test both foliar and root applications of AgNPs was based on common agricultural practices for fungicide application. While Foc is a root pathogen, fungicides are often applied either as a foliar spray or as a drench to the roots. By testing both methods, we aimed to explore whether AgNPs could be effective in controlling the pathogen through either approach. We have addressed this point in the discussion section. (See line 432 - 442)

**Comment 9:**
"AgNPs affected in different forms the study strains." (line 199)
**Response:**
We agree with the suggestion. We restructured the text: "The study strains showed different sensitivities to the AgNPs." on line 230 - 231. This difference is due to the sensitivity levels of each strain to the same AgNP.

**Comment 10:**
"~~Results of the percentages of~~" (line 202)
**Response:**
We agree with the suggestion.

**Comment 11:**
"~~4 strains~~" (line 202)
**Response:**
We agree with the suggestion.

**Comment 12:**
"Please improve the figure quality, this description is repetitive, it is included in the Fig. A, B and C are not necessary." (line 205)
**Response:**
We agree with the comment. We changed the figure description to "**Growth inhibition of Foc race 1 at different AgNP concentrations for three AgNP samples**. Bar diagrams show the effect of the AgNP samples on Fusarium inhibition growth" on line 235, and deleted the labels A, B, and C from Figure 1. The quality of the figure was improved as well.

**Comment 13:**
"discussion" (line 209)
**Response:**
We agree with this comment. In response, we restructured the idea to report the results "and the $IC_{50}$ value for the EC35-G-GM6 strain was a value of 7.39 mg $L^{-1}$", and the idea was discussed on line 361 - 364.

**Comment 14:**
"Try to use this description structure in Fig. 1." (line 217)
**Response:**
We agree with this comment. We used the writing style from this description to improve Figure 1 description.

**Comment 15:**
"Discussion" (line 221)
**Response:**
We agree with this comment. We added this line in the discussion section on line 363-364.

**Comment 16:**
"„the rest of treatments..." (line 224)
**Response:**
We agree with this comment. We made the change to "the rest of the treatments" on line 259.

**Comment 17:**
"greenhouse" (line 242)
**Response:**
We agree with this suggestion. We made the change to "Greenhouse bioassays" on line 276.

**Comment 18:**
"methodology" (line 243)

**Response:**
We agree with this suggestion. We deleted that line because it was already explained in the methodology on lines 206 - 214.

**Comment 19:**
"a higher" (line 250)
**Response:**
We agree with this suggestion. It was changed in the manuscript the expression "a better" for "a higher" on line 285.

**Comment 20:**
"than" (line 251)
**Response:**
We agree with this suggestion. It was changed in the manuscript the expression "to" for " than" on line 287.

**Comment 21:**
"Use a table format" (line 255)
**Response:**
We agree with this suggestion. We added a table format to Table 2 on line 294.

**Comment 22:**
"add this to the title" (line 257)
**Response:**
We agree with this suggestion. We restructured the title of Table 2 "Table 1. Results of the means values of Foc race 1 interaction with each AgNP in the greenhouse bioassay." on line 291 - 292.

**Comment 23:**
"The" (line 260)
**Response:**
We agree with this comment. We changed the expression "In relation to" for "The relation to" on line 296.

**Comment 24:**
"provides" (line 260)
**Response:**
We agree with this comment. In the manuscript, we changed the word "provide" to "provides" in line 296.

**Comment 25:**
"methodology" (line 262)
**Response:**
We agree with this suggestion. We added this part to the methodology on line 176 - 180.

**Comment 26:**
"was" (line 267)

**Response:**
We agree with this comment. W changed the word "can" to "was" on line 303.

**Comment 27:**
"~~Argovit-1220, Argovit-1221, and Argovit-C~~" (line 268)
**Response:**
We agree with this suggestion.

**Comment 28:**
"this is discusssion" (line 268)
**Response:**
We accept the suggestion. We added that line to the discussion section of the Antifungal activity of AgNPs in greenhouse conditions on line 410 - 411.

**Comment 29:**
"by Foc race 1" (line 271)
**Response:**
We agree with the comment. We added to the description of Figure 5 "Fusarium wilt by Foc race 1" on line 308 and 316 (Fig. 5 and 6).

**Comment 30:**
"Please check (all pape) if "infection" is correct..... It is more adecuated inoculation???" (line 272)
**Response:**
We accept the suggestion. The term 'infection' has been replaced with "inoculation" throughout the text to ensure accuracy.

**Comment 31:**
"Methodoloy and introduction" (line 287)
**Response:**
We agree with this suggestion. We deleted this sentence because it was already explained in the previous sections of the manuscript.

**Comment 32:**
"Conclusion" (line 288)
**Response:**
We agree with the comment. We placed the idea in conclusion on line 489 - 491.

**Comment 33:**
"Introduction" (line 290)
**Response:**
We agree with the comment. We added this line to the introduction section on lines 78 and 79.

**Comment 34:**

"~~Interestingly~~" (line 314)
**Response:**
We agree with the suggestion.

**Comment 35:**
"of who" (line 315)
**Response:**
In response, we clarified in the manuscript on line 363 the specific strain that presents the lowest susceptibility. The updated text is: "indicating that it had the lowest susceptibility to the AgNPs compared to the other strains tested."

**Comment 36:**
"Introduction" (line 322)
**Response:**
We accept the suggestion. We added the text to the introduction section on lines 89 - 95 for more consistency.

**Comment 37:**
"Results, please focus the discussion of the current results. The opportunities they imply" (line 329)
**Response:**
We appreciate the insightful suggestion. In response, we expanded the discussion of the results and the potential opportunities they present, emphasizing the significance of the study on lines 395 - 401 of the manuscript.

**Comment 38:**
"Methodology" (line 341)
**Response:**
We agree with the comment. We have removed this text, as it was redundant and had already been explained in the methodology

**Comment 39:**
"Results" (line 343)
**Response:**
We agree with the comment. We have removed this text, as it was redundant and had already been explained in the results.

**Comment 40:**
"~~in the experiment of the present work~~" (line 356)
**Response:**
We agree with the suggestion.

**Comment 41:**
"~~It is important to~~" (line 357)
**Response:**
We agree with the suggestion.

**Comment 42:**
"~~good~~" (line 368)
**Response:**
We agree with the suggestion.

**Comment 43:**
"positive" (line 368)
**Response:**
We agree with this comment.

**Comment 44:**
"or in a practical both methods can be integrated" (line 370)
**Response:**
We agree with this comment. We restructured the text and integrated the reviewer's idea. The updated text is: "In practice, both foliar and root application methods can be integrated to enhance efficacy" on lines 442 – 443.

**Comment 45:**
"Please develop more this concept... this is an opportunity to enforce the study core." (line 373)
**Response:**
We appreciate this suggestion. We expanded the discussion and enforced the study core on lines 449-456.

**Comment 46:**
"~~We did not find any publication in the literature dedicated to the study *in vivo* assay of the use of AgNPs against *Fusarium* species~~" (line 380)
**Response:**
We agree with the suggestion.

**Comment 47:**
"the novelty of this study..." (line 382)
**Response:**
We agree with the suggestion. We simplified the idea by adding "The novelty of this study" as it was suggested on line 465.

**Comment 48:**
"~~be an excellent alternative~~" (line 386)
**Response:**
We agree with the suggestion.

**Comment 49:**
"repetition" (line 393)
**Response:**
We agree with the suggestion. We removed that part from the conclusion section as it was redundant in the manuscript.

**Comment 50:**
"~~It is important to emphasize that we did not find any research conducted on inhibiting *Fusarium oxysporum* f.sp. *cubense* race 1, whether *in vitro* or *in vivo*. So, according to our best knowledge~~" (line 406)
**Response:**
We agree with the suggestion.

**Reviewer 2:**
**Comment 1:**
"in a greenhouse" (line 35)
**Response:**
We agree with the suggestion. We clarified in the abstract by adding "greenhouse conditions" to better explain the in vivo bioassay used in this study, as noted on lines 35-36.".

**Comment 2:**
"oKK, Clear" (line 94)
**Response:**
We appreciate the comment.

**Comment 3:**
"then harvest, and photo of the corm damage per plant??" (line 171)
**Response:**
We agree with the suggestion. We added that part to the methodology on lines 202-203.

**Comment 4:**
"Figure of Fig." (line 193)
**Response:**
We're not entirely sure what is meant by this comment. Could you please clarify what you mean by 'Figure of Fig'?

**Comment 5:**
"Improve the quality." (Figure 1)
**Response:**
We appreciate the suggestion. In response, we improved the quality of Figure 1.

---

**Review Comments to the Author**

**Reviewer #1:**
**Comment 1:**
The last paragraph in the Introduction Section should be the objective of this study.

**Response:**
We agree with reviewer #1 and restructure the sentence to: "In the present study, our main goal was to assess the inhibitory effect of AgNPs over Fusarium wilt on bananas" on line 97.

**Comment 2.**
"What are the specific VCGs of those Race 1 used in this experiment?"
**Response:**
VCGs were added in the methodology section on line 107.

**Comment 3.**
"How many plants per replicate are used in each treatment?"
**Response:**
We have addressed this concern in the methodology section on lines 180 to 182, where we explain the experimental setup and plant distribution. The experimental variables included the type of AgNP types of applications (foliar or drench), the two inoculation methods (root dipping or drenching), and the three AgNP samples. This resulted in the following treatment groups: foliar–root dipping–sample1, foliar–root dipping–sample2, foliar–root dipping–sample3, foliar–drenching–sample1, foliar–drenching–sample2, foliar–drenching–sample3, drench–root dipping–sample1, drench–root dipping–sample2, drench–root dipping–sample3, drench–drenching–sample1, drench–drenching–sample2, and drench–drenching–sample3. Each treatment had 5 replicates, for a total of 60 plants. For the positive controls, we used 12 plants (6 for root dipping and 6 for drenching), and for the negative controls, we used 12 plants as well (6 for foliar and 6 for drench application).

**Comment 4.**
"Linking to AgNPs mode of action, any measurements to provide a direct proof?"
**Response:**
The study focused on evaluating the inhibitory effect of silver nanoparticles through both in vitro assays and in vivo assays under greenhouse conditions. Negative and positive controls were used to quantify this effect and assess the potential of the nanoparticles. The modes of action of the silver nanoparticles were not evaluated for several reasons: first, it was not the objective of the study; second, an existing study performed at the University of China has already assessed the mode of action of silver nanoparticles against *Fusarium oxysporum*, it is cited in the paper. It showed that they primarily act by damaging the cell wall and membrane, also producing reactive oxidative species, disrupting the cellular metabolism, and causing damage in the cytoplasmic and organelle. Additionally, the limitations of our laboratory did not allow for experiments involving electron microscopy to investigate this aspect further. However, we have addressed this point in the discussion section to acknowledge the limitation. (See lines 342-349)

**Comment 5.**
"How about AgNPs physiological effects on banana plants? Any plant growth parameter measurements after 40 days experiment?"
**Response:**
No physiological effects on plants were evaluated in the study. However, in parallel with this experiment, another experiment was developed to evaluate the effects of AgNPs when applied to banana plants at different AgNPs concentrations. However, this research is in the

process of being published. According to the results of the effects study, something interesting that we were aware of is that AgNPs are distributed systemically throughout the plant, including by foliar application. Therefore, we were confident that the concentration used in our study (100 mg $L^{-1}$) would not cause phytotoxic effects on the plant.

**Comment 6.**
"Add biosafety issue of AgNPs in agricultural application"
**Response:**
We agree with the suggestion. We add to the discussion some of the concerns raised about the application of AgNPs in agricultural practices. (see lines 471-480)
**Reviewer #2:**

**Comment 1:**
"Dear Authors. This is an innovative research to show alternative options to face Fusarium wilt. The found data gives the apportunity of making a very impresive paper. However, the division between the sections (introductin, MM, Results...) must be standed. In some cases the sections are combined ahd there is a lack in discussion about the novelty of the results and their practical applications."
**Response:**
Thank you very much for your insightful suggestion. We have reorganized the writing to ensure that each section contains relevant information, and we have also added more details discussing the future application of AgNPs as a fungicide alternative. We hope our revisions meet the reviewers' expectations for publication.

---

We sincerely appreciate the thoughtful feedback provided by the reviewers, which has significantly improved the quality of our manuscript. We hope that the revised version meets the expectations of the reviewers and yourself. Please do not hesitate to contact us if further clarifications are needed.

Thank you for considering our manuscript for publication in PLOS ONE.

Sincerely,

Firmado electrónicamente por:
PABLO ANTONIO CHONG AGUIRRE

Pablo Chong Ph.D.
On behalf of all authors
