# Supplementary material for: Inhibition of Fusarium oxysporum growth in banana by silver nanoparticles: In vitro and in vivo assays
Source: PLoS One. 2025 Feb 24;20(2):e0308200. doi: 10.1371/journal.pone.0308200 (PMC11849816; doi:10.1371/journal.pone.0308200)
Supplement: Attachment — Submitted filename: Response to reviewers.docx [file pone.0308200.s005.docx]

Dear Adalberto Benavides-Mendoza, Ph.D.,

Academic Editor,

PLOS ONE

We express our sincere gratitude for your thoughtful consideration and constructive feedback on our manuscript, **Inhibition of *Fusarium oxysporum* growth in banana by silver nanoparticles: *in vitro* and *in vivo* assays** submitted to **PLOS ONE.** We appreciate the time and effort you have taken to review our work and provide suggestions.

In response to the editor's comments, we have made the following revisions:

- Ensure that our manuscript meets PLOS ONE's style requirements, including those for file naming.
- State what role the funders took in the study and include the amended Role of Funder statement in our cover letter.
- Remove the funding information from the Acknowledgments section or other areas of our manuscript.
- Upload a copy of Figures 1 to 6.
- Include captions for our Supporting Information files at the end of our manuscript.
- All relevant data (raw data) of the in vitro and in vivo assays has been shared as a supporting information compressed file name Raw Data.7z

We hope that the revisions we have made satisfactorily address the reviewers' concerns. We are open to making any additional changes that may be required. Thank you once again for your time and consideration.

Sincerely,

Pablo Chong Ph.D.

On behalf of all authors
